# Supplementary material for: IMPACT smoking cessation support for people with severe mental illness in South Asia (IMPACT 4S): A protocol for a randomised controlled feasibility trial of a combined behavioural and pharmacological support intervention
Source: PLoS One. 2023 Jun 14;18(6):e0287185. doi: 10.1371/journal.pone.0287185 (PMC10266604; doi:10.1371/journal.pone.0287185)
Supplement: S1 File — (DOCX) [file pone.0287185.s002.docx]

**IMPACT smoking cessation support for people with severe mental illness in South Asia (IMPACT 4S): a protocol for a randomised controlled pilot and feasibility trial for a combined behavioural and pharmacological support intervention**

##### University of York, UK:

Dr Noreen Mdege (workstream co-lead)

Dr Gerardo Zavala

Dr Papiya Mazumdar

Prof Kamran Siddiqi

Dr Najma Siddiqi

Prof Catherine Hewitt

Prof Simon Gilbody

##### National Institute of Mental Health and Neurosciences (NIMHANS), Bangalore, India:

Prof Pratima Murthy (workstream co-lead and country lead)

Dr Krishna Prasad

Prof Santosh Kumar Chaturvedi

Dr Arun Kandasamy

##### Institute of Psychiatry, Rawalpindi, Pakistan:

Dr Faiza Mujeeb (country lead)

Prof Asad Nizami

Dr. Ba ha

##### University of Leeds, UK:

Dr Ian Kellar

##### Valid Research UK:

Dr Cath Jackson

##### London School of Economics, UK:

Assoc Prof David McDaid

**Summary**

| Title | IMPACT Smoking cessation support for people with severe mental illness in South Asia (IMPACT 4S): a protocol for a randomised controlled pilot and feasibility trial for a combined behavioural and pharmacological support intervention |
| --- | --- |
| Short Title | IMPACT 4S pilot and feasibility trial |
| Version | V 1.3 |
| Date | 22/03/2021 |
| ISRCTN | ISRCTN34399445 |
| Study design | Randomised controlled pilot and feasibility trial |
| Study duration | 36 months (twenty-four months adaptation, nine months recruitment + follow-up, three months data analysis and report writing) |
| Population | Adults with severe mental illness (i.e. schizophrenia, schizoaffective disorder, bipolar affective disorder, psychosis, severe depression with psychosis) |
| Setting | Mental health facilities in India and Pakistan |
| Treatment(s) | *Brief advice*: five minutes, face-to-face, one-to-one smoking cessation counselling session and an information leaflet.  *Combined behavioural and pharmacological support intervention (IMPACT 4S intervention)*: The intervention comprises of up to 15 one-to-one and/or remotely delivered smoking cessation counselling sessions, each lasting between 15-40 minutes and spread over three/four months, breath carbon monoxide monitoring and feedback, pharmacotherapy (bupropion and/or nicotine replacement therapy), and an information leaflet. Remote sessions can be delivered using a number of different options such as telephone or video platforms (e.g. What’s App, messenger, Zoom etc.). |
| Study aim | To adapt an evidence based, combined behavioural and pharmacological support intervention for smoking cessation among people with severe mental illness; and test the feasibility of delivering and evaluating it in India and Pakistan. |
| Study outcome(s) | Primary outcomes (feasibility and acceptability)  1. Recruitment rates: Quantitative assessment of the acceptability of the research will be assessed by numbers screened, number eligible and those agreeing to participate. 2. Reasons for ineligibility/non-participation/non-consent of participants. 3. Length of time required to achieve the required sample size. 4. Retention in study: Assessed as a proportion of those enrolled in the study who are successfully followed-up at six months. 5. Retention in treatment: Evaluated by number of study intervention sessions attended as one measure of the feasibility and acceptability of the trial interventions to participants. 6. Intervention fidelity during the delivery of the behavioural support within the IMPACT 4S intervention, as well as for brief advice (BA), assessed as one measure of feasibility of intervention delivery. 7. Smoking cessation pharmacotherapy adherence: For those in the IMPACT 4S arm, adherence to smoking cessation pharmacotherapy will be assessed as one measure of the feasibility and acceptability of the smoking cessation pharmacotherapies to participants. 8. Data completeness: Data will be checked for completeness as another measure of acceptability and feasibility of data collection methods, and to identify problem areas and solutions.  Secondary outcomes  1. Self-reported or family/carer reported continuous smoking abstinence for at least six months (only five instances of smoking allowed during the total six months) which is biochemically verified by CO concentration (CO concentration <7ppm) at six months follow-up. This will be assessed at the longest possible follow-up point for those participants where it might not be possible to have a six months follow-up. 2. Point abstinence, defined as a self-report or family/carer report of not smoking in the previous 7 days, assessed at one, three and six months follow-up. This will be assessed at the longest possible follow-up point for those participants where it might not be possible to have a six months follow-up. 3. Cost of delivering the IMPACT 4S and the BA interventions. |
| Number of participants | 172 in total (86 in India; 86 in Pakistan) |
| Main inclusion/ exclusion criteria | Inclusion Criteria  - Adults (≥18 years old) with SMI (i.e. schizophrenia, schizoaffective disorder, bipolar affective disorder, psychosis, severe depression with psychosis) - Considered to be stable by the mental health clinical team - Self-reported current smoker of any form of smoked tobacco product (including cigarettes, bidis, waterpipe etc) for at least 6 months - Smoking on >25 days in the past month - Able to provide informed consent - Attending / remotely accessing services from included institutions during the study period - Willing to cut down or quit smoking - Willing and able to attend up to 15 face-to-face and/or remotely delivered counselling sessions - Living in the Rawalpindi district in Pakistan, and in Bangalore urban and rural, nearby/neighbouring districts in India.  Exclusion Criteria  - Pregnant or breastfeeding women - Comorbid drug and alcohol problems |
| Estimated period of recruitment | 6 months |
| Total duration per participant | 6 months |
| Statistical methodology | Quantitative analysis will focus on descriptive summaries. For each group the numbers of individuals approached, randomly assigned, receiving intended treatment, completing the study protocol, and providing outcome data will be summarised.  The number of individuals withdrawing from the intervention and/or the trial, and where available, the reasons for withdrawal, will be summarised by trial arm.  For each data collection point the number of non-responders will be calculated and attendance rates compared.  A CONSORT diagram will be provided to display the flow of participants through the study. |
| Sponsor | University of York |
| Funder | National Institute of Health Research [Grant reference number 17/63/130] |
| Principal Investigators | Professor Pratima Murthy- NIMHANS, Bangalore, India  Dr. Noreen Mdege- University of York, UK  Professor Simon Gilbody- University of York, UK |

Contents

[1. Introduction 6](#_Toc66101199)

[2. Study aims 7](#_Toc66101200)

[2.1. Overall aim 7](#_Toc66101201)

[2.2. Objectives 7](#_Toc66101203)

[2.3. Research questions 7](#_Toc66101204)

[3. Overall study design 8](#_Toc66101206)

[4. Intervention Adaptation 8](#_Toc66101208)

[5. Feasibility testing 8](#_Toc66101209)

[5.1. Details of the interventions 9](#_Toc66101210)

[5.2. Study sites and settings 11](#_Toc66101211)

[5.3. Study Population 11](#_Toc66101212)

[5.4. Eligibility 11](#_Toc66101213)

[5.5. Recruitment 12](#_Toc66101214)

[5.6. Eligibility assessment 12](#_Toc66101215)

[5.7. Consent 12](#_Toc66101216)

[5.8. Withdrawals 13](#_Toc66101217)

[5.9. Reimbursements 13](#_Toc66101218)

[5.10. Randomisation procedure 13](#_Toc66101219)

[5.11. Trial arm allocation 13](#_Toc66101220)

[5.12. Blinding 14](#_Toc66101221)

[5.13. Measures to avoid/ minimising bias 14](#_Toc66101222)

[5.14. Study outcomes 14](#_Toc66101223)

[5.15. Baseline and follow-up data collection 15](#_Toc66101224)

[5.16. Frequency and duration of follow-up 16](#_Toc66101225)

[5.17. Data collection and entry 18](#_Toc66101226)

[5.18. Sample size and statistical analysis 19](#_Toc66101227)

[5.19. Analysis of economic and quality of life data 19](#_Toc66101228)

[6. PROCESS EVALUATION 20](#_Toc66101229)

[6.1. Implementation (feasibility) and context 20](#_Toc66101230)

[6.2. Implementation (fidelity) 20](#_Toc66101231)

[6.3. Mechanisms of impact (acceptability and mediators) 20](#_Toc66101232)

[7. DATA MANAGEMENT 21](#_Toc66101233)

[7.1. Data handling and storage 21](#_Toc66101234)

[7.2. Archiving 21](#_Toc66101235)

[8. DATA MONITORING 21](#_Toc66101236)

[9. OTHER ETHICAL CONSIDERATIONS 22](#_Toc66101237)

[10. ADVERSE EVENTS 22](#_Toc66101238)

[10.1. Description of potential adverse events 22](#_Toc66101239)

[10.2. Adverse event monitoring 23](#_Toc66101240)

[10.3. Definitions 24](#_Toc66101241)

[10.4. Trial site responsibilities 25](#_Toc66101242)

[10.5. Notification procedure 27](#_Toc66101243)

[10.6. Country PI/ lead responsibilities 27](#_Toc66101244)

[10.7. University of York research team responsibilities 28](#_Toc66101245)

[10.8. Guidance for stopping the trial 28](#_Toc66101246)

[11. incidental findings 28](#_Toc66101247)

[12. STUDY MANAGEMENT STRUCTURE 29](#_Toc66101248)

[12.1. Study management group 29](#_Toc66101249)

[12.2. Independent Trial Steering Committee (ITSC) 29](#_Toc66101250)

[12.3. Independent Data Monitoring and Ethics Committee (DMEC) 29](#_Toc66101251)

[13. Study Timeline 30](#_Toc66101252)

[14. REFERENCES 30](#_Toc66101253)

## Introduction

The rapidly increasing burden of mental disorders now accounts for one in every 10 years of health lost globally.^1,2^ People with severe mental illness (SMI)(schizophrenia, bipolar disorder, other psychoses and severe depressive disorder) constitute one of the world’s most vulnerable populations,^3^ facing huge health and socioeconomic inequalities, with twice the mortality^3^ and 10-25 years lower life expectancy than the general population.^4^ This is principally attributable to coexisting physical disorders,^5^ due to adverse lifestyle choices, such as smoking, alcohol use, diet, illicit drug use, poor diet and physical inactivity.^6^ Global health policies and research agendas have, therefore, prioritised addressing the physical health of people with SMI,^7^ with an emphasis on interventions targeting health risk behaviours.^8^

Tobacco use is the leading modifiable contributor to morbidity and mortality worldwide, causing seven million deaths: approximately six million as a result of direct tobacco use, of which 270,000 are from smokeless tobacco use; and around 890 000 as a result of non-smokers being exposed to second-hand smoke.^9,10^ Annually tobacco use results in the loss of 45.9 million disability adjusted life years (DALYs) worldwide: around 35 million DALYs are lost as a result of direct tobacco use, of which 6.4 million DALYs lost are from smokeless tobacco use;^11,12^ and 10.9 million DALYs are lost due to second hand smoke exposure.^9,10^ Global tobacco-related deaths are expected to reach more than eight million by 2030;^13^ 80% of these deaths will be in low and middle income countries (LMICs),^14^ with half occurring in working age adults, causing significant loss to economic productivity.^15^

South Asian countries have some of the highest rates of tobacco use.^16,17^ For example, in India 28.6% of all adults (42.4% of men and 14.2% of women) use some form of tobacco; with the smoking prevalence being approximately 10% for all adults (19% for men and 2% for women).^18^ For Pakistan, about 19.1% of all adults (32% of men and 5.8% of women) use some form of tobacco, with the smoking prevalence being 12.4% overall among adults (22.2% among men and 2.1% among women).^19^ 21.4% (29.6% for men and 12.8% for women) and 7.7% (11.4% of men and 3.7% of women) of the adult population in India and Pakistan, respectively, use smokeless tobacco.^18,19^

Tobacco use prevalence is particularly high in people with SMI, with estimates ranging from 50-90%.^20^ Specifically, smoking prevalence among SMI is estimated to range from 40 to 60%.^20,21^ Limited data from South Asian studies suggest smoking prevalence as high as 50% among people with SMI.^21^ People with SMI also smoke more heavily, have more severe nicotine dependence, and face worse health outcomes as a result of tobacco.^22^ Although smoking is one of the most common modifiable unhealthy behaviours among people living with SMI,^23^ benefits of smoking cessation programmes have not been extended to this population. There has been almost no reduction in smoking prevalence among people with SMI, even in high income countries (HICs), despite the dramatic reduction in smoking rates in the general population that has occurred during the past 40 years.^23^ The reasons for this include “diagnostic overshadowing” whereby physical health is neglected in the presence of a mental illness diagnosis; and the unsuitability of interventions designed for the general population that are predicated on high levels of cognitive function, literacy, health literacy, motivation and self-efficacy, all of which may be compromised by SMI.^6,24^ For example, for the general population, individual counselling has been shown to increase the chance of quitting by between 40% and 80%, compared to minimal support,^25^ whilst group support has been shown to increase the chances of quitting by 88% compared to self-help programme.^26^ For people with SMI however, there has been mixed results from low quality studies in HIC. Some studies have concluded behavioural support interventions were effective but others have found that they do not increase the chances of quitting smoking among adults with SMI.^27-29^ Nevertheless, available HIC evidence to date suggests that pharmacotherapies alone (bupropion and varenicline),^30^ or in combination with behavioural support^31^ are safe, effective and acceptable for smoking cessation in adults with SMI.

Tailored smoking-cessation interventions that take account of some of the challenges among people with SMI have been developed for HICs^29^ (although the evidence-base remains limited), but not for LMICs. Differing patterns of tobacco use, diet, health service provision, and regulatory approaches mean that evidence from an HIC cannot be directly translated to a LMIC. Interventions targeting South Asian countries would need to take into account the high prevalence of smoking. The cultural perceptions of smoking may also differ between Western countries and countries in South Asia. In addition, many of the currently existing smoking-cessation studies among people with SMI have been judged to be low quality in general,^29,32^ and this needs to be strengthened.

## Study aims

### Overall aim

To adapt an evidence based, combined behavioural and pharmacological support intervention (hereafter referred to as the IMPACT 4S intervention) for smoking cessation among adults with SMI attending mental health facilities in India and Pakistan, and test the feasibility and acceptability of delivering and evaluating it.

###

### Objectives

1. To adapt an evidence based intervention (IMPACT 4S) for smoking cessation for people with SMI.
2. To test the feasibility and acceptability of delivering the IMPACT 4S intervention for smoking cessation among people with SMI
3. To test the feasibility and acceptability of carrying out a definitive trial to evaluate the effectiveness and cost-effectiveness of the IMPACT 4S intervention for smoking cessation among people with SMI

### Research questions

#### Delivering the intervention (for both face-to-face and remote modes)

1. What is the feasibility of delivering the IMPACT 4S intervention for smoking cessation in people with SMI?
2. What is the acceptability of the IMPACT 4S intervention among people with SMI who are smokers and psychology graduates and social workers who deliver the intervention?
3. What adaptations need to be made to the IMPACT 4S intervention before it is evaluated in a definitive trial?

#### Recruitment, Randomisation and Retention

1. What are the overall recruitment, enrolment and attrition rates?
2. What are the attrition rates per trial arm?

#### Identification of measures for data collection

1. What is the feasibility and acceptability of collecting data using the proposed tools and methods for baseline and follow-up variables at one, three and six months?

What is the feasibility and acceptability of measuring breath carbon monoxide (CO) levels for biochemical validation of smoking cessation status?

## Overall study design

The study consists of three main components: i) intervention adaptation that will last for six months; ii) pilot and feasibility trial (including the collection of effectiveness and cost-effectiveness data) that will last for 12 months; and iii) process evaluation embedded within the feasibility trial period. These are described below.

##

## Intervention Adaptation

The intervention will be based on three behavioural support intervention manuals from the SCIMITAR+,^33^ and TB & Tobacco^34^ trials, as well as the “Tobacco use: A Smart Guide” which is a quitting program developed by the National Institute of Mental Health and Neuro Sciences Resource Centre for Tobacco Control Bangalore, India.^35^ We will use an iterative co-design approach comprising 3-5 intervention development workshops with smoking cessation specialists, behavioural scientists, policymakers, practitioners and people with SMI aged 18 or over who smoke. The workshops will identify the processes through which behaviour change occurs (mechanisms of action [MOAs]),^9,36,37^ and behavioural change techniques (BCTs) that are important for changing smoking behaviour among adults with SMI who smoke^38^ using the information gathered from rapid reviews of literature and the views of smoking cessation experts within our study group. MOAs and BCTs will be chosen based on perceived strength of association with smoking cessation and modifiability within this population. The workshops will also be used to refine the modes of delivery for the intervention. Following the workshops, the research team will further expand the selected MOAs, BCTs, modes of delivery into “core” intervention modules (e.g. problem-solving) and manuals that are potentially applicable.

We will then hold two consultation workshops per country with people with SMI who smoke where we will present our intervention modules and seek their views on the acceptability of the intervention modules, activities, key messages, and materials, and ideas for improving them, particularly in terms of making them culturally relevant for each of the two countries. The findings will be utilised to refine the intervention manuals and modules.

## Feasibility testing

This is a parallel open label randomised, controlled pilot and feasibility trial in which participants will be allocated 1:1 to the following two arms:

- Arm 1: Brief advice
- Arm 2: IMPACT 4S intervention

### Details of the interventions

#### Arm 1: Brief advice

Participants randomised to trial arm 1 will receive brief advice (BA) adapted from a community-based trial of tobacco cessation in India.^39^ As part of BA, participants will receive verbal information on the harmful effects of tobacco and will be advised to stop smoking. BA will be delivered by trained psychology graduates in Pakistan and psychology graduates and social workers in India on the same day as enrolment and will last up to five minutes. This will be a one-time interaction: participants in this trial arm will not have any further BA sessions with the psychology graduates and social workers. Participants will be provided with an information leaflet containing the same advice in a written format to take home.

#### Arm 2: IMPACT 4S intervention

The participants randomised to trial arm 2 will receive a behavioural support intervention adapted during the intervention adaptation phase, breath CO monitoring and feedback, pharmacotherapy (bupropion and/or nicotine replacement therapy), and the same information leaflet as for the BA arm to take home.

The behavioural support will include BCTs chosen from a BCT taxonomy from a European context as no similar taxonomy has yet been developed for smoking cessation in LMICs^40^ and will also include BCT previously used for smoking cessation India.^41^ The BCTs will include goal setting, problem solving, social support, information about health consequences and adding objects to the environment. Participants will be encouraged to: (1) reduce smoking to quit, (2) set their own quit dates and (3) make several attempts to quit if their initial attempt fails. The IMPACT 4S intervention has been designed for either face-to-face, remote, or a combination of both face-to-face and remote sessions, to allow adaptation to practical feasibility and participants’ preferences. Remote sessions can be delivered using a number of different options such as telephone or video platforms (e.g. What’s App, messenger, Zoom etc.). The IMPACT 4S Intervention comprises of up to 15 one-to-one counselling sessions, each lasting between 15-40 minutes duration. Each session will be delivered by trained psychology graduates in Pakistan and psychology graduates and social workers in India. Delivery will be facilitated through the use of an intervention manual/guide, a flipbook containing key messages on how to quit smoking, and participants’ resource material. In the case of face-to-face delivery, the manual or flipbook will help intervention delivery staff to deliver messages via photo images on the front of the slides and text on the back of slides and same messages will be reiterated in posters and leaflets. This type of intervention delivery involving face-to-face mode is familiar for health and social workers as well and counsellors in these settings and has been found feasible and acceptable in the TB & Tobacco trial being conducted in Bangladesh and Pakistan.^34^ In order to facilitate the remote delivery of the intervention: 1) an adapted remote intervention manual will be use to deliver the intervention in a way that is consistent with face-to-face sessions; and 2) participants will be supplied with a set of seven-colour coded information sheets in advance that will provide information for each session, including any relevant ‘tasks’ that the participant will be required to complete for each session (e.g. smoking diary, reviewing the checklist for quit day and noting down reasons for stopping.

In addition, participants in this arm will get breath CO monitoring and feedback on their breath CO levels during face-to-face counselling session. CO monitoring is a valuable motivational tool for smokers. Research has shown that smokers are more likely to make a successful quit attempt if a CO breath monitor is used as part of a supported and structured quit plan.^42,43^ In case of remote delivery when CO breath monitoring is not possible, CO readings would be discussed at every appointment during remote delivery in the same way as they would for face to face. The only difference being, instead of carrying out a carbon monoxide reading, advisors would give example readings of what they would expect to see based on the patient’s level of smoking or progress in quitting smoking.

Participants in this arm will also be offered pharmacological support to aid smoking cessation (nicotine replacement therapy or bupropion either separately or ideally in combination). Pharmacotherapies will be provided for a minimum period of three months. Participants who opt to take bupropion will be referred to their medical doctor for assessing suitability of prescribing bupropion. Participants will be offered sustained-release bupropion, 150 mg/d for the first week and 300 mg/d thereafter. The pharmacological support will be provided for free within the trial. The participants will be scheduled for prescribed medication collection during their face-to-face sessions. In case of changes in mobility restrictions as complying to the local government guideline for managing the upcoming Covid-19 pandemic situation, all scheduled prescribed medication shall be dispatched or personally delivered to the participants’ residential address by post or by research assistants, respectively. Medications for one month will be handed over to them personally by the research staff. An excel sheet log will be maintained for the dispensation, consumption, replenishing and monitoring of Bupropion/Nicotine gums for all the participants who choose pharmacotherapy. The excel sheet log will be filled for medication related details by the research assistants delivering the 4S intervention to the participants and will be centrally managed by the trial manager. The trial manager and the research assistant will monitor the excel sheet log daily to verify if patients would be close to finishing their one-month’s stock of Bupropion/Nicotine gums and need replenishment of a fresh stock. Patient participants who are close to finishing their medications will be given a telephone call one week in advance and asked to visit the NIMHANS campus again to collect the smoking cessation medication for another month; in case, they cannot come to collect the medications, the medications will be personally delivered to the patients. The medications will be provided for a period of three months in total. If there is requirement of bupropion/nicotine gums beyond three months, they will be referred to the treating clinical unit. The patients will be reimbursed the travel costs and compensated for the time they spent in procuring the medication from NIMHANS. The patients will be educated about the correct dosage and method of use, side effects of bupropion/nicotine gums along with an information leaflet on the medications and will be provided contact details to report side effects and for advice. A referral to emergency services will be facilitated in case of a serious adverse event. The participants will be informed to keep the medications in their safe custody and out of reach of children. bupropion/nicotine gums are medications that are routinely prescribed for smoking cessation, including for persons with severe mental illness. Suicide risk assessment will be carried out during the sessions in the intervention arm. Consent to contact family members in case the patient is not reachable on telephone will be sought to mitigate any risks associated with overdose. In case of overdose, a referral to the nearest emergency services will be facilitated. The adherence to medications will be documented in the counsellor’s record book in each session.

The psychology graduates and social workers with similar levels of training and experience on smoking cessation interventions will deliver the behavioural support in the IMPACT 4S intervention for and the brief advice for people on both arms. The people delivering the interventions will receive relevant training on the intervention they will be delivering. The duration of training will be half a day for the brief advice intervention, and one to two days for the IMPACT 4S intervention. During the intervention delivery period, the psychology graduates and social workers delivering trial interventions will also receive ongoing support and supervision from the experienced team members.

### Study sites and settings

In India the feasibility trial will be conducted at the National Institute of Mental Health and Neurosciences (NIMHANS), Bangalore, a tertiary care neuropsychiatric institute. In Pakistan the feasibility trial will be conducted at the Institute of Psychiatry (IoP), Rawalpindi.

### Study Population

Outpatients with SMI seeking care in either of the participating mental health institutions.

### Eligibility

#### Inclusion Criteria

- Adults (≥18 years old) with SMI (i.e. schizophrenia, schizoaffective disorder, bipolar affective disorder, psychosis, severe depression with psychosis)
- Considered to be stable by the mental health clinical team
- Self-reported current smoker of any form of smoked tobacco product (including cigarettes, bidis, waterpipe etc) for at least 6 months
- Smoking on >25 days in the past month
- Able to provide informed consent
- Attending / remotely accessing services from included institutions during the study period
- Willing to cut down or quit smoking
- Willing and able to attend up to 15 face-to-face and/or remotely delivered counselling
- Living in the Rawalpindi district in Pakistan, and in Bangalore urban and rural districts in India

#### Exclusion Criteria

- Pregnant or breastfeeding women
- Comorbid drug and/or alcohol problems

### Recruitment

Patients from NIMHANS, Bangalore, India and IoP, Rawalpindi, Pakistan who are diagnosed with SMI (schizophrenia, schizoaffective disorder, psychosis, bipolar disorder, depression with psychotic symptoms) and are current smoked tobacco users, will be referred to the study team by their care team. A screening form based on the eligibility criteria (*see section 5.4.*) will be used to identify eligible participants.^44^ All recruitments to be done through face-to-face mode.

Participants from the IMPACT survey (i.e., a currently ongoing survey led by members of our study team and gathering data on the physical comorbidities and lifestyle-risk behaviours, including smoking, among people with SMI in India and Pakistan), who are smokers and have agreed to be contacted for future studies, will also be contacted and asked if they wish to participate in the study. In such a case the recruitment will happen remotely due to the restrictions imposed by the pandemic, i.e., on telephone or on video-call based on the convenience of the participant. The process of obtaining informed consent and the verbal consent provided will be audio/video recorded in such instances.

### Eligibility assessment

Each participant will be allocated a unique screening number. In addition, the following anonymised individual information will be collected and stored in a secure study database: age; gender; SMI diagnosis; current smoking status; eligibility criteria; and time taken for screening. For those identified as not being eligible for the study, the reason for exclusion will also be recorded.

Individuals who meet the eligibility criteria will be invited to participate in the study. They will receive a detailed study information sheet including the following information: the purpose of the feasibility study; the study procedures including details of interventions, process of randomisation, the collected information and the frequency and timing of data collection, breath CO monitoring; the potential benefits or risks of participating in the trial; information on privacy and confidentiality of the participants’ data, and how it will be processed; and participant’s rights, including voluntary nature of the trial and the right to discontinue participation at any time in the trial without any consequences. The information sheet will also provide contact details of a research team member in case they wish to seek more information or clarity. In the circumstance that a potential participant is unable to read or write, the researcher will provide facilitation by reading out the information sheet to both the participant and the carer if present and will respond to their queries. Participants will be given 24 hours to discuss the information with family members or friends before making a decision if they require it.

### Consent

Informed consent will be obtained before any study specific baseline assessments. Once the participants are familiar with the intent and purposes of the study, and have clarified their queries with the researcher, those willing to participate will be invited to provide written consent. For the face-to-face recruitment, we will follow a written informed consent procedure where each participant will provide their full name and signature or, if they cannot write, a thumb impression (with a witness present) as allowable according to the ethics requirements. We will follow a formal informed consent procedure where each participant will provide their full name and signature or, if they cannot write, a thumb impression (with a witness present) as allowable according to the ethics requirements in India and Pakistan. One copy of the consent form will be securely stored in a locked filing cabinet at the trial sites in India and Pakistan, separate from the rest of the trial data. Each study participant will get a copy of their signed informed consent form, and the information sheet to take home for their records. For participants who would be recruited remotely, a researcher will first read out the participant information sheet to sensitize them adequately about the purpose, procedures involved in the 4S study, participant roles and their rights, and other important ethical aspects. Afterwards, a consent form will be read out to each participant confirming their awareness about the study, their voluntary participation, confidentiality, compensation matters, and usage of the research data. Having confirmed their understanding and awareness of the study, their verbal consent will be sought, and audio/video recorded.

All consenting individuals will be registered in a secure study database, using their name, age, address, and unique study ID (*see section 5.10. for the procedure for allocating study IDs*). Only the principal investigators, country investigators, trial coordinators, and researchers involved in collecting, quality checking and entering data will have access to these identifiable data at any stage of the trial mainly to facilitate participant follow-up. Identifiable data will be stored separate to the baseline and follow-up data. For the purpose of baseline and follow-up data collection and conducting analyses, only the unique study IDs will be used, thereby ensuring anonymity of data.

### Withdrawals

Participants will be free to withdraw consent and leave the trial at any time without giving a reason. Information (written/verbal) on who to contact if they wish to withdraw from the trial will be provided to all participants. Written information on who to contact if they wish to withdraw from the trial will be provided to all participants. They will be able to withdraw by letting any member of the research team know that they wish to do so. If a participant withdraws consent to participate, no further data will be collected from them. However, data collected up to the point of withdrawal will be retained and used in the analysis, except where withdrawal of consent for the use of this data is explicit, in which case all data will be destroyed.

### Reimbursements

Participants will not be offered any personal incentive for taking part in the trial. However, at every visit for receiving the 4S intervention session or collecting bupropion/nicotine gums, each trial participant will receive reimbursement of actual travel cost. In addition, they will receive INR 250 (India)/PKR 500 (Pakistan) as compensation for time spent. The amount of reimbursement will be reviewed by the local/National Ethics Review Boards as per local prerequisites.

### Randomisation procedure

Those eligible participants who consent will be allocated a study ID and randomly assigned to one of the trial arms using a computer-generated blocked stratified (by country) randomisation sequence created using Stata version 15 (or later) with an allocation ratio of 1:1. A statistician based at the University of York, who is not involved in the recruitment of trial participants, will generate the randomisation sequence and produce the opaque sealed envelopes as detailed below.

### Trial arm allocation

Participants will be allocated to trial arms using opaque sealed envelopes stored securely at the central research office. After a participant has provided written informed consent, a researcher will make a phone call to the country specific trial manager who will be based at the central research office. Upon being provided with basic participant information, the trial manager will allocate the next envelope in the sequence to the participant. Up until this point, both the trial manager and the research officer will be unaware of the trial arm associated with the envelope. After opening the envelope, the trial manager will indicate to the researcher whether the participant will receive the IMPACT 4S intervention or brief advice and the envelope number and allocation will be recorded on the participant record. For the participants who are recruited remotely, the researcher may inform them on the same day about results of trial arm allocation over phone if they agree to be contacted again on the same day. If the participant did not agree to be contacted again on the same day, they will be informed about the trial arm allocation on a mutually agreed upon day and date for the next telephonic call.

### Blinding

Blinding the participants and clinicians from knowing who is receiving the IMPACT 4S intervention or BA is not feasible. Outcome data collection and data analysis will also not be fully blinded.

### Measures to avoid/ minimising bias

The process of randomisation will minimise the chance of selection bias. In order to minimise loss to follow-up, participants will be requested to inform the trial team of any relevant changes, for example if they plan to relocate or their contact details change. Researchers will also be in regular contact with the participants via the phone. Participants will be informed about follow-up prior to the date. Information materials in the appropriate language will be provided to ensure participants understand clearly what the expectations would be if they decide and give consent to participate in the trial.

### Study outcomes

The study will focus on the following primary (feasibility and acceptability) and secondary outcomes.

#### Primary outcomes

1. Recruitment rates: Quantitative assessment of the acceptability of the research will be assessed by numbers screened, number eligible and those agreeing to participate.
2. Reasons for ineligibility/non-participation/non-consent of participants.
3. Length of time required to achieve the required sample size.
4. Retention in study: Assessed as a proportion of those enrolled in the study who are successfully followed-up at six months.
5. Retention in treatment: Evaluated by number of study intervention sessions attended as one measure of the feasibility and acceptability of the trial interventions to participants.
6. Intervention fidelity during the delivery of the behavioural support within the IMPACT 4S intervention, as well as for BA, assessed as one measure of feasibility of intervention delivery.
7. Smoking cessation pharmacotherapy adherence: For those in the IMPACT 4S arm, adherence to smoking cessation pharmacotherapy will be assessed as one measure of the feasibility and acceptability of the smoking cessation pharmacotherapies to participants.
8. Data completeness: Data will be checked for completeness as another measure of acceptability and feasibility of data collection methods, and to identify problem areas and solutions.

#### Secondary outcomes

1. Self-reported or family/carer reported continuous smoking abstinence for at least six months (only five instances of smoking allowed during the total six months) which is biochemically verified by CO concentration (CO concentration <7ppm) at six months follow-up.^45^ This will be assessed at the longest possible follow-up point for those participants where it might not be possible to have a six months follow-up.
2. Point abstinence, defined as a self-report or family/carer report of not smoking in the previous seven days, assessed at one, three and six months follow-up. This will be assessed at the longest possible follow-up point for those participants where it might not be possible to have a six months follow-up.
3. Cost of delivering the IMPACT 4S and the BA interventions.

### Baseline and follow-up data collection

Data will be measured and collected from participants and carers face-to-face or remotely at baseline, one, three and six months. Data will be either be entered in paper format or directly into an online survey tool (Qualtrics)^46^ using tablets.

#### Demographic data (baseline)

The information on age and gender collected at the participant eligibility screening stage will be verified by asking the participant. We will also collect data on education, occupation, marital status, and on the possession of a number of household items to determine socio-economic status following the demographics module of the STEPs WHO questionnaire.^47^

#### Psychiatric medication use (baseline, one, three and six months)

Details of psychiatric medication that the participant is taking will be taken at baseline, and verified at each follow-up visit, in-order to capture any changes.

#### Smoking status (baseline, one, three and six months)

Self-reported or family/carer reported smoking status will be assessed using the smoking questions from the GATS survey.^48^ At six months, self-reported smoking status will be biochemically verified by a CO level of <7ppm for those reporting that they have quit smoking, as per the Russell Standard.^45^ When a participant self-reports abstinence with an elevated CO level of >7ppm, the biochemical verification will supersede the self-report and the participant will be defined as a smoker. The CO assessment for biochemical verification of smoking status cannot be done in case of remote recruitment of trial participants or remote delivery of the 4S intervention to the trial participants

#### Nicotine dependence and urge to smoke (baseline, one, three and six months)

The heavy smoking index will be used to assess nicotine dependence from smoked tobacco.^49^ The Mood and Physical Symptoms Scale (MPSS)^50^ will also be administered. The scale assesses withdrawal symptoms including anxiety, depression, irritability, restlessness, hunger, concentration and sleep.

#### Quitting smoking intentions, motivations and behaviors (baseline, one, three and six months)

The four item motivation to quit (MTQ) questionnaire^33^ will be used to measure motivation to quit smoking. It is scored by summing the responses to each item. The scores range from 4 to 19 where a higher score indicates greater motivation to quit. Questions from the Global Adult Tobacco Survey (GATS)^48^ will also be used to assess smoking cessation intentions, motivations and behaviours.

#### Mediators of smoking cessation (baseline, three and six months)

A set of questions related to the interventions’ behaviour change targets will be developed during the intervention adaptation phase and used to assess the mediators of smoking cessation in this study.

#### Quality of life (baseline, three and six months)

The EQ-5D-5L^51,52^ will be used to measure health-related quality of life (HRQoL). EQ-5D is a standardised measure of health status developed by the EuroQol Group in order to provide a simple, generic measure of health for clinical and economic appraisal, where health is characterised on five dimensions (mobility, self-care, ability to undertake usual activities, pain/discomfort, anxiety/ depression).

#### Health services use (baseline, three and six months)

Participant use of health services and other smoking cessation services outside the study will be assessed. A health service utilisation questionnaire previously used in some of our studies such as the MCLASS trials^53^ or the Client Service Receipt Inventory, adapted to the context of Pakistan and India,, will be used to collect number and type of contacts with doctors, hospital admissions, pharmacy visits and medication prescriptions for all participants. Information on contact with traditional healers will also be recorded.

#### Mental and physical health (baseline, three and six months)

PHQ-9^54^ will be used to measure depressive symptoms. This nine item questionnaire is scored from 0 to 27, and a higher score indicates more severe depressive symptoms. The GAD-7^55^ will be used for measuring anxiety. This seven-item instrument is scored from 0 to 21, with a higher score indicating more severe anxiety. The SF-12 which consists of two subscales: a physical health component and a mental health component will also be administered. Both components are scored from 0 to 100, with 0 indicating the lowest level of health and 100 the highest level of health measured by the scale.^56^

#### Physical body measurements (baseline, three and six months)

Physical body measurements will include height and weight which will be measured by trained personnel according to the WHO protocols.^57^ These will be used to determine the body mass index. Physical body measurements of the trial participants will not be measured if the recruitment and assessments are conducted remotely, or the intervention is done delivered remotely.

#### Smoking cessation pharmacotherapy adherence (one and three months)

For the IMPACT 4S group, self-reported medication adherence to smoking cessation pharmacotherapies will be assessed retrospectively based on a 4-day recall.^58^

An adherence index will be calculated by the formula:

Total number of doses taken X 100

Total number of doses prescribed

Participants with more than 80% of adherence will be considered as having high adherence and those with less than 80% will be considered as having low adherence.^59^ Participants who report missed doses will be asked to provide reasons for missing their medications.

- - 1. Participants’ preference between face-to-face and remote delivery of intervention for those who are on the IMPACT 4S intervention arm (at baseline)

Participants will be asked to indicate their preferred mode of intervention delivery (face-to-face or remote), and the reasons for their preference.

- - 1. Carers/ family members involvement (at baseline)

Participants will be asked to indicate whether they had been accompanied by a carer or family member at any of the intervention session, and whether they received help with any of the intervention activities from a carer/ family member.

### Frequency and duration of follow-up

Following randomisation and baseline data collection, participants in each arm will be followed up at one, three and six months post-randomisation. At each follow-up visit, a trained researcher will administer a structured follow-up data collection form to the trial participant, obtaining information according to the follow-up schedule below (Table 1). The study flow diagram is given in Figure 1 below.

**Table 1. Baseline and follow-up measurement**

| **Measurement** | **Baseline** | **1 month**  **(Follow-up 1)** | **3 months**  **(Follow-up 2)** | **6 months**  **(Follow-up 3)** |
| --- | --- | --- | --- | --- |
| Demographic data | X |  |  |  |
| Preference for mode of intervention delivery | X |  |  |  |
| Carers/ family members’ involvement | X |  |  |  |
| Details of psychiatric medication | X | X | X | X |
| Smoking status | X | X | X | X |
| Nicotine dependence and urge to smoke | X | X | X | X |
| Quitting smoking intentions, motivations and behaviours | X | X | X | X |
| Mediators of smoking cessation | X |  | X | X |
| Health-related quality of life | X |  | X | X |
| Mental and physical health | X |  | X | X |
| Health services use | X |  | X | X |
| Physical body measurements (height; weight) | X |  | X | X |
| Smoking cessation pharmacotherapy adherence |  | X | X | X |
| Process evaluation questionnaire and interviews |  |  | X |  |

**Figure 1. Trial scheme diagram**


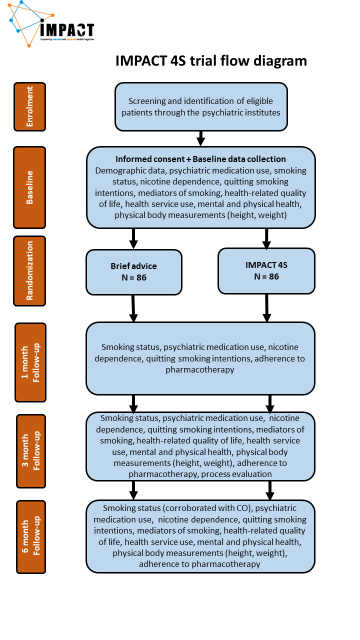


### Data collection and entry

Researchers in India and Pakistan will screen and recruit participants, and collect baseline and follow up information at one, three and six months. These researchers will receive three days training on trial procedures including taking informed consent, administering and completing the questionnaires and ethical issues such as autonomy of individual participants on making decisions about participation, freedom to withdraw from the trial without giving any reason or consequence, privacy, confidentiality, anonymity.

Data will be entered either in paper format or directly into an online survey tool (Qualtrics), ^46^ a secure and password protected resource available at the University of York. Collected data will be stored on a central database server.

All data will be stored and transferred following Health Insurance Portability and Accountability Act protocol. Any transfer of data from study sites to the University of York will be via the University of York’s secure and encrypted Drop-Off service. The staff involved in the trial will be trained on data protection processes. The staff will be strictly monitored to ensure compliance with privacy standards.

### Sample size and statistical analysis

#### Sample size

This will be an external feasibility trial and the main purpose is to explore the feasibility of the new intervention and the methods of recruitment, randomisation and follow up for a full trial. The sample size calculations are based on estimating participation and attrition rates and event rates of the primary outcome. If we identify 344 eligible subjects we will be able to estimate a participation rate of 50% to within a margin of error of ±5% and an attrition rate of 30% to within ±8%.^60^ Furthermore, an external feasibility study of at least 120 measured participants will provide robust estimates of event rates to inform the sample size calculation for the subsequent larger definitive fully powered trial.^61^ We will therefore aim to enrol 172 (86 per country) participants in the study in order to retain 120 for analysis assuming an attrition rate of 30%.

#### Statistical analysis

Quantitative analysis will focus on descriptive summaries. For each group the numbers of individuals approached, randomly assigned, receiving intended treatment, completing the study protocol, and providing outcome data will be summarised.  The number of individuals withdrawing from the intervention and/or the trial, and where available, the reasons for withdrawal, will be summarised by trial arm.  For each data collection point the number of non-responders will be calculated and attendance rates compared.  A CONSORT diagram will be provided to display the flow of participants through the study.

### Analysis of economic and quality of life data

The completeness of returned HRQoL and health care utilisation data will be assessed to inform an economic evaluation alongside a full RCT. Returned service use data will be used to revise questionnaires used in a full trial.

Costs of delivering the interventions will be measured by recording the resources utilised in the delivery of the IMPACT 4S intervention (including behavioural support, CO monitoring and feedback, pharmacotherapy, costs for telephone/remote modes, client information-sheets, advisor’s resource for clients’ records and information leaflet costs) and BA (including the counselling and information leaflet). We will also capture the costs of intervention setup, training, as well as ongoing support and supervision of the personnel delivering the intervention. We will capture participant travel costs and travel time to attend intervention sessions, including whether they were accompanied by carers/families, and the related travel and time costs. For each trial arm, the costs of providing the intervention (including set-up, training, and ongoing support and supervision) will be calculated. Given the sample size, we would not conduct a full economic evaluation at this stage, as a much larger RCT is needed to undertake a cost-effectiveness analysis.

**Suicidality:** If during the recruitment, assessments or intervention delivery, the participant expresses thoughts, feelings or other or signs that indicate threat to his or her life, appropriate action will be taken to handle it. SoPs for dealing with low, medium and high risk would be as follows:

***Low risk:*** The patients with Low risk will be recommended for Tele follow up (IVRS- Interactive voice response system) and will be linked with the concerned unit for needed intervention

***Medium and High risk:*** The patient with medium and high risk will be advised to get admitted in emergency. If the patient permits/consents family members will be informed

about the patients heightened risk, information on monitoring and treatment.

## PROCESS EVALUATION

The process evaluation will be informed by the Medical Research Council guidance for process evaluation^62^ which identifies three components: implementation, mechanisms of impact and context.

### Implementation (feasibility) and context

Once delivery of the IMPACT 4S and brief advice interventions are completed, the psychology graduates and social workers who delivered these interventions will be interviewed to explore their experiences, including the acceptability of the interventions and the barriers and drivers to delivery including contextual factors such as the health facility environment.

### Implementation (fidelity)

Fidelity to delivering the behavioural support and brief advice interventions will be assessed using a fidelity index. This consists of two sub-indices: The Adherence Index that assesses adherence to the intervention activities; and the Quality Index, that assesses competence with which the intervention was delivered. Both indices are scored on a three-point Likert scale (0=not implemented, 1=partially implemented and 2=fully implemented).

All counselling sessions will be audio-recorded. The researchers will perform the fidelity check by reviewing the audio-recording the counselling sessions of a randomly selected 10% of participants per trial arm. For the IMPACT 4S group, they will ensure a mix of participants who completed/did not complete all their sessions.

### Mechanisms of impact (acceptability and mediators)

To capture the views and experiences of participants, all participants will complete a short questionnaire at the end of the intervention, as part of the three month follow up. Depending on their trial arm allocation, this will explore which components of the brief advice/ IMPACT 4S intervention they engaged with, the acceptability of each session including the CO monitoring, their use of pharmacotherapies and perceived impact of the interventions on their smoking behaviour. Participants in both trial arms will complete some acceptability questions on trial processes.

A purposive sample of participants in each country (12-16 in the IMPACT 4S arm, 4-8 in the BA arm), reflecting a mix of men and women who have/have not quit smoking will be interviewed to collect more in-depth feedback. For the IMPACT 4S intervention, this will be 3-4 participants each for men who quit, women who quit, men who did not quit, women who did not quit; whilst for BA it will be 1-2 participants for each group.

Data on potential mediators likely to influence the mechanism of impact of the intervention ingredients on cessation will be collected at all follow-ups (*see 5.15.6*).

All interviews will be conducted face-to-face using a topic guide and digitally audio-recorded. They will be about an hour long. A hermeneutics approach, which encourages participants to discuss features of the intervention to elicit data on their experience and evaluation of its delivery/receipt will be used.^63^

Participants will provide written informed consent for these interviews, separate from the trial, after having received enough information for this study component and their questions being answered. The consent procedures will be the same as for the trial.

The quantitative data from the fidelity index and questionnaire will be analysed using descriptive statistics, including means and standard deviation for continuous variables, and absolute and relative frequencies for categorical variables. Interviews will be transcribed verbatim and translated into English and analysed using the Framework Approach^64^ which is designed to address applied programme and policy-related questions. NVivo 11 software will aid data handling. Integration of interview findings with respective questionnaire data will be done using a ‘triangulation protocol’.^65^

## DATA MANAGEMENT

### Data handling and storage

The researchers will assure that participants’ anonymity is maintained and that their identities are protected from unauthorised parties. Participants will be assigned unique study IDs and these will be used on all data collection questionnaires, tools and other study records; participants will not be identified by their names. The country coordinating centres will keep and maintain any study logs (including screening and enrolment logs) showing study IDs, names, age and other identifiable information of the participants securely under password protection (for electronic records) or lock and key (if hard copies). The study sites have filing cabinets with lock and key for data storage. All consent forms will be secured safely in a separate compartment of this cabinet. Study information (including consent forms, screening and enrolment data, baseline and follow-up data, audio-recordings and transcripts) will only be accessed by researchers, and will not be released without written permission, except as necessary for monitoring by study monitors or regulatory bodies, for example if the study is audited. Encrypted data transfer between the study sites and the University of York will be via secure Drop-off services available through the University of York.

### Archiving

At the end of the study, data will be securely archived by the University of York, NIMHANS and IoP, as appropriate, for 10 years.

## DATA MONITORING

Data will be monitored for quality and completeness by a delegated researcher at the study sites, followed by a second check by the study Research Fellow at the University of York using verification, validation and checking processes. We will follow the York Trials Unit’s guidance regarding processes for data monitoring. Missing data will be pursued until study end unless they are data obtained by participant contact and this would cause distress. Data will be reported to the Independent Trial Steering Committee (ITSC) and Data Monitoring and Ethics Committee (DMEC) as required.

## OTHER ETHICAL CONSIDERATIONS

The study will commence following a formal ethics review and approval from the University of York’s Health Sciences Research Governance Committee. Ethics applications will also be submitted to the National (Indian Council of Medical Research, National Bioethics Committee Pakistan) and where required, institutional (such as the Ethics Committee (Behavioural Sciences Division), NIMHANS, Bangalore, India) levels in all participating India and Pakistan.

This study is part of a programme of research in South Asia called IMPACT. Approvals will also be sought collectively for the IMPACT studies from each administrative site in each participating country prior to commencement of IMPACT’s research activities.

The study will adhere to the fundamental principles of human rights and dignity laid down in the Declaration of Helsinki. Every possible effort will be made to uphold the principles of autonomy, justice, beneficence and non-maleficence in the design and conduct of the study. Firstly, the eligibility criteria are broad and inclusive to ensure inclusion of groups that may benefit from the trial. There will be no monetary benefits for recruited trial participants. Individuals will provide an informed consent and will have the right to discontinue participation at any point in the study. During study visits and data collection, efforts to minimize inconvenience and burden to participants will be maximised including seeking prior appointments for home visits if these are necessary. Enrolled participants will always be assured of complete confidentiality of their data; participant names will not be used.

## ADVERSE EVENTS

The principles of ICH GCP require that both investigators and sponsors follow specific procedures when notifying and reporting adverse events or reactions in clinical trials. This section of the protocol describes the potential adverse events (AEs), adverse event monitoring during the trial, definitions of adverse events and reporting procedures.

### Description of potential adverse events

Below we describe the potential AEs from each of the components of the study.

#### Feasibility trial

Research procedures

Our procedures for questionnaire completion involve interviewer-administration where participants will be asked questions that they might find uncomfortable to answer. All our study procedures will be conducted by experienced and appropriately qualified researchers who are trained on privacy and confidentiality. These researcher will provide assurance to participants that any information provided will be kept confidential, and study data will only contain thier study ID and not participant names or any other information that might be used to identify the respondents. Whilst answering all questions would really benefit the study achieve its aims and objectives, there might be questions that participants might feel strongly that they do not want to answer. We will advise them to let the researcher know so that they do not continue to ask them those specific questions. It is possible that some of the questions might cause distress. The participants will be advised to ask the researcher to take a break or stop the interview all together. The researcher will also seek help from clinic staff (e.g. counsellors) on the participant’s behalf if the participant feels they need such help.

Trial interventions

Behavioural support, information leaflet and CO monitoring: These are low risk interventions. However, in some circumstances the information or feedback provided might cause anxiety and distress for participants. In such circumstances, researcher will seek help from clinic staff (e.g. counsellors) on the participant’s behalf if the participant feels they need such help.

Pharmacological support: Bupropion and nicotine replacement therapies are generally safe medicines, and have very few side effects. For bupropion, these include nausea, stomach ache, dryness of mouth, sleeplessness, mood changes, appetite changes and headache. We will seek advice from the participant’s mental health care team on whether they can take bupropion before we prescribe this for them. Nicotine gum gives small amounts of nicotine through a gum; and the patches will deliver it through the skin. While the participant will continue to get some nicotine in their system, they will not be exposed to any of the other harmful chemicals that are found in tobacco. Some of the side effects from nicotine gum are mouth or throat sores, bad aftertaste, problems with existing dental work, nausea, jaw pain and racing heartbeat. Some of the side effects of nicotine patches are skin irritation, itching, dizziness, headache, racing heartbeat and nausea. We will advise participants about these potential side effects, and to contact the study team as soon as possible if they suffer from any side effects (serious or otherwise), for us to advise them on what to do. If the side effect(s) are serious enough and the participant feels they need immediate medical attention, they will be informed that they should seek medical help in such circumstances and inform the medical team about their participation in the study and the medicines they are taking as part of the study, and also other medicines that they might be taking.

#### Process evaluation

Our process evaluation component mainly involves interviews with participants and the psychology graduates/social workers that will deliver the interventions to explore their experiences with the intervention. Although we do not foresee any concerning harms from this, our researchers will receive training in supporting interviewees in case of any distress; a break may be taken in the interview, help may be sought from clinic staff (e.g. counsellors) or the interview can be resumed at a later date or cancelled altogether.

### Adverse event monitoring

AEs will be assessed at each counselling session and at the face-to-face follow-up visits, and their potential association with study interventions or procedures ascertained. The researcher conducting the counselling session or follow-up visit assessments will record all directly observed AEs and all AEs reported by the study participant. An adverse events review checklist will be developed to be used at each visit / session to explicitly prompt for symptoms relating to possible nicotine replacement therapy or bupropion related toxicities. This way we will be able to address any AEs or concerns that the participants might have.

In the context of this study, it is very unlikely that any AEs would be related to the behavioural support within the IMPACT 4S intervention nor the BA as they are educational and motivational intervention and that are not harmful. The research procedures are also very low risk. Nevertheless, there is a potential for AEs due to the pharmacotherapies (nicotine replacement therapy or bupropion). AEs will be reported on the AE form. Serious adverse events will be defined according to ICH GCP, and will be reported to the respective country coordinating centres according to the agreed timelines.

### Definitions

Table 2 below shows the definitions of AEs that will be used in the study.

**Table 2. AE definitions**

| **Term** | **Definition** |
| --- | --- |
| Adverse Event (AE) | Any untoward medical occurrence in a participant to whom an IMP has been administered including occurrences that are not necessarily caused by or related to that product. |
| Adverse Reaction (AR) | Any untoward and unintended response to an IMP related to any dose administered. |
| Unexpected Adverse Reaction (UAR) | An adverse reaction, the nature or severity of which is not consistent with the information about the medicinal product in question set out in the SPC for that product, participant information leaflet, IB or protocol. |
| Serious Adverse Event (SAE) or Serious Adverse Reaction (SAR) or Suspected Unexpected Serious Adverse Reaction (SUSAR) ^***^ | Respectively any adverse event, adverse reaction or unexpected adverse reaction that:   - Results in death - Is life-threatening^*^ - Requires hospitalisation or prolongation of existing hospitalisation^**^ - Results in persistent or significant disability or incapacity - Consists of a congenital anomaly or birth defect |

^*^The term life-threatening in the definition of a serious event refers to an event in which the participant is at risk of death at the time of the event; it does not refer to an event that hypothetically might cause death if it were more severe, for example, a silent myocardial infarction.

^**^Hospitalisation is defined as an inpatient admission, regardless of length of stay, even if the hospitalisation is a precautionary measure for continued observation. Hospitalisations for a pre-existing condition, that has not worsened or for an elective procedure do not constitute an SAE.

^***^ Medical judgement should be exercised in deciding whether an AE or AR is serious in other situations. The following should also be considered serious: important AEs or ARs that are not immediately life-threatening or do not result in death or hospitalisation but may jeopardise the participant or may require intervention to prevent one or the other outcomes listed in the definition above.

#### Adverse events

AEs include:

- An exacerbation of a pre-existing illness
- An increase in frequency or intensity of a pre-existing episodic event or condition
- A condition (even though it may have been present prior to the start of the trial) detected after trial medication administration
- Continuous persistent disease or a symptom present at baseline that worsens following administration of the study treatment

#### Exempted adverse events

AEs do not include:

- Medical or surgical procedures; the condition that leads to the procedure is the adverse event
- Pre-existing disease or a condition present before treatment (i.e. a disorder present at the baseline study visit and noted on the baseline medical history/physical examination form/medical notes) that does not worsen
- Hospitalisations where no untoward or unintended response has occurred, e.g., elective cosmetic surgery, social admissions
- Overdose of medication without signs or symptoms

### Trial site responsibilities

Participants will be advised to record the nature, timing and duration of any AEs with clear guidance on when to stop the medication and when and how to report back to the named clinician at the participating mental health facilities. These clinicians will have received the basic training required in this regard. All non-serious AEs and ARs, whether expected or not, should be recorded in the participant’s medical notes and recorded in the study AE Form by the researcher and sent to the country trial manager/ coordinator within an agreed timescale (preferably three days)- the details will be provided in the ‘Adverse Event and Serious Adverse Event Reporting’ standard operating procedure (SOP). SAEs and SARs should be notified to the country lead (a medical doctor) within 24 hours of the researcher/clinical team becoming aware of the event.

#### Trial site assessment

Seriousness

When an AE or AR occurs, the clinical team responsible for care of the participant must first assess whether or not the event is serious using the definition given in Table 2. If the event is serious, then an SAE/SUSAR Form must be completed and the country lead or trial manager notified within 24 hours.

Severity or Grading of Adverse Events

This relates to the intensity or severity of the event experienced by the participant. The severity of all AEs and/or ARs (serious and non-serious) in this trial should be based on the clinical team’s (supported by the country lead or trial manager if needed) judgement using the following definitions:

- Mild: An event that is easily tolerated by the participant, causing minimal discomfort and not interfering with everyday activities.
- Moderate: An event that is sufficiently discomforting to interfere with normal everyday activities.
- Severe: An event that prevents normal everyday activities.

*NOTE: Severity should not be confused with the seriousness of the event which relates to the participant or event outcome.*

Causality

The country leads or mental health care team must assess the causality of all serious events or reactions in relation to the trial therapy using the definitions in Table 3. Alternative causes, such as natural history of the underlying diseases, concomitant therapy, other risk factors etc. should be considered. There are five categories: unrelated, unlikely, possible, probable, and definitely related. If the causality assessment is unrelated or unlikely to be related, the event is classified as an SAE. If the causality is assessed as possible, probable or definitely related, then the event is classified as an SAR.

**Table 3: Assigning Type of SAE through Causality**

| **Relationship** | **Description** | **SAE type** |
| --- | --- | --- |
| Unrelated | There is no evidence of any causal relationship | Unrelated SAE |
| Unlikely | There is little evidence to suggest that there is a causal relationship (for example, the event did not occur within a reasonable time after administration of the trial medication). There is another reasonable explanation for the event (for example, the participant’s clinical condition, other concomitant treatment). | Unrelated SAE |
| Possible | There is some evidence to suggest a causal relationship (for example, because the event occurs within a reasonable time after administration of the trial medication). However, the influence of other factors may have contributed to the event (for example, the participant’s clinical condition, other concomitant treatments). | SAR |
| Probable | There is evidence to suggest a causal relationship and the influence of other factors is unlikely. | SAR |
| Definitely | There is clear evidence to suggest a causal relationship and other possible contributing factors can be ruled out. | SAR |

Expectedness

If there is at least a possible involvement of the trial treatment, the country lead or mental health care team must assess the expectedness of the event. An unexpected adverse reaction is one not previously reported in the current Summary of Product Characteristics or one that is more frequent or more severe than previously reported. The definition of an unexpected adverse reaction (UAR) is given in Table 2. The ‘Adverse Event and Serious Adverse Event Reporting’ SOP will contain a list of expected toxicities associated with the medications being used in this trial. If a SAR is assessed as being unexpected, it becomes a SUSAR.

Notification

The country lead or trial manager should be notified of all SAEs **within 24 hours** of the site’s mental health care team or researcher becoming aware of the event. The mental health care team should notify country lead or trial manager of all SAEs occurring from the time of randomisation until 30 days after the last protocol treatment administration using the SAE/SUSAR Form. SARs and SUSARs must be notified to the country trial managers or country leads until trial closure.

### Notification procedure

1. The SAE/SUSAR Form must be completed by the site clinician (who is responsible for the participant’s care), with due care being paid to the grading, causality and expectedness of the event as outlined above. In the absence of the responsible clinician, the form should be completed and signed by a suitably qualified member of the mental health care team or the trial researcher and sent to the country office as appropriate. The responsible clinician should subsequently check the SAE/SUSAR Form; make changes as appropriate, sign and then re-send to the respective country coordinating centre as soon as possible. The initial report must be followed by detailed, written reports as appropriate.

The minimum criteria required for reporting an SAE are the study ID and age, name of clinician reporting, the event, and why it is considered serious.

1. The SAE/SUSAR Form must be sent as a scanned copy by email to the respective country coordinating centre:

| **SAE REPORTING**  Within 24 hours of becoming aware of an SAE, please fax or email a completed SAE form to the respective country office on:  India: Fax (To Be Advised), email: (To Be Advised)  Pakistan: Fax (To Be Advised), email: (To Be Advised) |
| --- |

1. Follow-up: participants must be followed up until clinical recovery is complete (including laboratory results returning to normal or baseline if relevant), or until the event has stabilised. Follow-up should continue after completion of protocol treatment if necessary. A further SAE/SUSAR Form, by indicating as ‘Follow-up report’ should be completed and emailed to the country coordinating centre as information becomes available. Extra, annotated information and/or copies of test results may be provided separately. The participant must be identified by study ID, age and initials only. The participant’s name should not be used on any correspondence and should be deleted from any test results.
2. Staff should follow their institution’s procedure for local notification (for e.g. to drug regulatory and Research Ethics Committees (REC)/ Institutional Review Boards (IRB)) requirements.

### Country PI/ lead responsibilities

Medically-qualified staff at the country coordinating centre (the country trial manger or lead) will review all SAE reports received. The events will be coded using the Medical Dictionary for Regulatory Activities ([MedDRA](http://www.meddra.org/)). The causality assessment given by the mental health care team at the mental health facility cannot be overruled; in the case of disagreement, both opinions will be provided in any subsequent reports.

The country leads are responsible for reporting the SAEs to the competent authorities (drug regulatory authorities and REC/IRB) in their respective countries and to the University of York research team in the UK. Fatal and life-threatening SUSARs must be reported to the competent authorities (drug regulatory authorities and REC/IRB) within 7 days of the country coordinating centre being notified of the event and follow-up information within a further 8 days; other SUSARs must be reported within 15 days.

The University of York research team should be notified of all SAEs **within 24 hours** of the site mental health care team’s becoming aware of the event. The country leads should notify the University of York research team of all SAEs occurring from the time of randomisation until 30 days after the last protocol treatment administration using the SAE/SUSAR Form. SARs and SUSARs must be notified to the University of York research team until trial closure.

### University of York research team responsibilities

The University of York research team is undertaking the duties of trial Sponsor and must ensure the reporting of SUSARs and other SARs, by the country leads, to the local competent authorities of the countries in which the trial is taking place and the REC/IRB, has been done appropriately.

The University of York research team will also keep all country leads informed of any safety issues that arise during the course of the trial.

#### Annual safety reports

The University of York research team, as delegated by the Sponsor, will submit Annual Safety Reports in the required format to the main ethics committee (Health Sciences Research Governance Committee- [HSRGC](http://www.york.ac.uk/healthsciences/research-information/rsg/)) which gave the favourable opinion 12 months after the date on which the favourable opinion was given and thereafter until the end of the study. This report will be provided to the country leads to submit to their national competent authorities.

#### Urgent safety measures

The country leads or trial managers may take appropriate urgent safety measures in order to protect research participants against any immediate hazard to their health or safety. These safety measures should be taken immediately and may be taken without prior authorization from the HSRGC or local competent authorities.

The country leads or trial managers must alert the University of York research team who must alert the sponsor as soon as possible of the urgent measures.

### Guidance for stopping the trial

The study will be stopped, as guided by the ITSC and DMEC, if:

- new literature indicates findings that can be applied to this question in terms of benefit or side effects. However, early evidence of clear benefit would not be a reason to halt recruitment in the trial.
- reporting of AEs indicate that review of the study protocol is required, for the study medication, or rescue medicine

## incidental findings

Our trial procedures present the potential of a number of incidental findings:

- At risk drinking, alcohol problems or problematic psychoactive substance use: Our screening procedure involves the collection and scoring of self-reported alcohol use and psychoactive substance use using the AUDIT and ASSIST Lite respectively. It is possible that some individuals will be identified as at-risk drinkers, individuals with alcohol problems or individuals with problematic psychoactive substance use. Our country research teams are experienced with dealing with these problems and work closely with addiction services. We will provide very brief information on the findings (including that this is not a diagnosis) and the harms of unhealthy drinking and a leaflet that is already used by the addiction services in-country. We will also refer the participant to the addiction services or other available services for further evaluation and treatment if required.
- Breath CO monitoring: It is possible for some breath CO readings to suggest potential CO poisoning from other sources. The participants will be informed of the findings and advised to seek immediate attention from their health service. The 4S intervention shall strive to minimize any risks for infection transmission by specifying required safety practices while handing CO-monitors. For the purposes of infection control, the bag containing the disposable mouthpiece should be tied up and placed in a second refuse bag, the machine should be cleaned between tests using a **non-alcohol sanitiser**. All advisors will need to sanitise hands (using a non-alcohol sanitiser) before and after every test. The CO monitoring will be done in a well aerated room and the participant will be instructed to blow into the mouthpiece in a direction away from the counsellor. The counsellors will be wearing N-95 masks and face-shields during the procedure. All counsellors shall be adequately trained and required to adhere to these safety guidelines throughout the period of intervention delivery.

## STUDY MANAGEMENT STRUCTURE

### Study management group

The Study Management Group (SMG) will oversee delivery of the project, guide the Research Fellows and other researchers involved in study implementation, and contribute to the write up and dissemination of findings. The SMG will consist of all of the above investigators.

### Independent Trial Steering Committee (ITSC)

An ITSC will be set up and will include an independent chair, independent statistician and at least two other independent members. The research team will also attend these meetings, particularly the co-PIs, country leads and the statistician. Other members of the team will also attend when required. The ITSC is likely to meet twice or three times during the lifespan of the trial, but the committee will decide on the frequency of meetings. The committee will provide an overall supervision of the trial and ensure that the study is conducted according to the protocol and within the overarching ethical framework through its independent chair. Members will also provide advice outside these meetings according to their area of expertise at key stages via e-mail, phone or if needed, face-to-face.

### Independent Data Monitoring and Ethics Committee (DMEC)

An independent DMEC will be formed, which will be the only group who sees the confidential, accumulating data for the trial. Reports to the DMEC will be produced by the study statisticians. The DMEC will meet within 6 months of the trial opening; the frequency of meetings will be dictated at the first meeting. The DMEC will consider data using the statistical analyses (*see Section 5.18*) and will advise the ITSC. The DMEC can recommend premature closure or reporting of the trial, or that recruitment to any trial arm is to be discontinued.

## Study Timeline

|  | 2019 | | | | 2020 | | | | 2021 | | | | 2022 | Milestones |
| --- | --- | --- | --- | --- | --- | --- | --- | --- | --- | --- | --- | --- | --- | --- |
| Activities | Q1 | Q2 | Q3 | Q4 | Q1 | Q2 | Q3 | Q4 | Q1 | Q2 | Q3 | Q4 | Q1 |  |
| Refinement of research objectives |  |  |  |  |  |  |  |  |  |  |  |  |  | Research objectives finalized |
| Study protocol writing |  |  |  |  |  |  |  |  |  |  |  |  |  | Study protocol (including registration) |
| Ethics and other approvals |  |  |  |  |  |  |  |  |  |  |  |  |  | Approval letters on file |
| Intervention development and adaptation phase (including adaptation to remote delivery) |  |  |  |  |  |  |  |  |  |  |  |  |  | Intervention manual; training manual |
| Recruitment |  |  |  |  |  |  |  |  |  |  |  |  |  |  |
| Feasibility testing and refining |  |  |  |  |  |  |  |  |  |  |  |  |  | Intervention ready definitive testing |
| Report writing |  |  |  |  |  |  |  |  |  |  |  |  |  | Final study report and manuscript |
| Grant proposal development |  |  |  |  |  |  |  |  |  |  |  |  |  | Grant proposal |
| Q1:jan-mar, Q2:apr-jun, Q3:jul-sep, Q3oct-dec | | | | | | | | | | | | | | |

## REFERENCES

1. Patel V, Chisholm D, Parikh R, et al. Addressing the burden of mental, neurological, and substance use disorders: key messages from Disease Control Priorities, 3rd edition. *Lancet.* 2016;387(10028):1672-1685.

2. Whiteford HA, Degenhardt L, Rehm J, et al. Global burden of disease attributable to mental and substance use disorders: findings from the Global Burden of Disease Study 2010. *The Lancet.* 2010;382(9904):1575-1586.

3. Walker ER, McGee RE, Druss BG. Mortality in mental disorders and global disease burden implications: a systematic review and meta-analysis. *JAMA psychiatry.* 2015;72(4):334-341.

4. Lawrence D, Kisely S, Pais J. The epidemiology of excess mortality in people with mental illness. *Canadian journal of psychiatry Revue canadienne de psychiatrie.* 2010;55(12):752-760.

5. Scott KM, Von Korff M, Alonso J, et al. Mental-physical co-morbidity and its relationship with disability: results from the World Mental Health Surveys. *Psychological medicine.* 2009;39(1):33-43.

6. De Hert M, Correll CU, Bobes J, et al. Physical illness in patients with severe mental disorders. I. Prevalence, impact of medications and disparities in health care. *World Psychiatry.* 2011;10(1):52-77.

7. Patel V, Saxena S, Frankish H, Boyce N. Sustainable development and global mental health—a Lancet Commission. *The Lancet.* 2016;387(10024):1143 -1145.

8. Ngo VK, Rubinstein A, Ganju V, et al. Grand Challenges: Integrating Mental Health Care into the Non-Communicable Disease Agenda. *PLoS medicine.* 2013;10(5):e1001443.

9. Connell LE, Carey RN, de Bruin M, et al. Links Between Behavior Change Techniques and Mechanisms of Action: An Expert Consensus Study. *Annals of behavioral medicine : a publication of the Society of Behavioral Medicine.* 2018.

10. World Health Organization. *WHO report on the global tobacco epidemic, 2017: Monitoring tobacco use and prevention policies.* Geneva: World Health Organization;2017.

11. Sinha DN, Suliankatchi RA, Gupta PC, et al. Global burden of all-cause and cause-specific mortality due to smokeless tobacco use: systematic review and meta-analysis. *Tobacco control.* 2018;27(1):35-42.

12. Reitsma MB, Fullman N, Ng M, et al. Smoking prevalence and attributable disease burden in 195 countries and territories, 1990–2015: a systematic analysis from the Global Burden of Disease Study 2015. *The Lancet.* 2017;389(10082):1885-1906.

13. Mathers CD, Loncar D. Projections of global mortality and burden of disease from 2002 to 2030. *PLoS Medicine.* 2006;3(11):e442.

14. World Health Organization. World No Tobacco Day 2017. 2017. <http://www.who.int/campaigns/no-tobacco-day/2017/event/en/>. Accessed 13 April 2018.

15. World Bank. Tobacco tax reform at the crossroads of health and development. 2017:12. <http://documents.worldbank.org/curated/en/491661505803109617/Main-report> Accessed 13 April 2018.

16. *WHO global report on trends in prevalence of tobacco smoking 2015* Geneva: World Health Organisation;2015.

17. Smoking prevalence and attributable disease burden in 195 countries and territories, 1990–2015: a systematic analysis from the Global Burden of Disease Study 2015. *The Lancet.* 2017;389(10082):1885-1906.

18. Tata Institute of Social Sciences (TISS), Mumbai and Ministry of Health and Family Welfare, Government of India. *Global Adult Tobacco Survey GATS 2 India 2016-17.* New Delhi: Ministry of Health and Family Welfare, Government of India;2018.

19. Saqib MAN, Rafique I, Qureshi H, et al. Burden of Tobacco in Pakistan: Findings From Global Adult Tobacco Survey 2014. *Nicotine & tobacco research : official journal of the Society for Research on Nicotine and Tobacco.* 2018;20(9):1138-1143.

20. Szatkowski L, McNeill A. Diverging trends in smoking behaviors according to mental health status. *Nicotine & tobacco research : official journal of the Society for Research on Nicotine and Tobacco.* 2015;17(3):356-360.

21. de Leon J, Diaz FJ. A meta-analysis of worldwide studies demonstrates an association between schizophrenia and tobacco smoking behaviors. *Schizophrenia research.* 2005;76(2-3):135-157.

22. Cook BL, Wayne GF, Kafali EN, Liu Z, Shu C, Flores M. Trends in smoking among adults with mental illness and association between mental health treatment and smoking cessation. *JAMA : the journal of the American Medical Association.* 2014;311(2):172-182.

23. Johnson JL, Ratner PA, Malchy LA, et al. Gender-specific profiles of tobacco use among non-institutionalized people with serious mental illness. *BMC psychiatry.* 2010;10(1):101.

24. Liu NH, Daumit GL, Dua T, et al. Excess mortality in persons with severe mental disorders: a multilevel intervention framework and priorities for clinical practice, policy and research agendas. *World Psychiatry.* 2017;16(1):30-40.

25. Lancaster T, Stead LF. Individual behavioural counselling for smoking cessation. *Cochrane Database of Systematic Reviews.* 2017(3).

26. Stead LF, Carroll AJ, Lancaster T. Group behaviour therapy programmes for smoking cessation. *The Cochrane database of systematic reviews.* 2017;3:Cd001007.

27. Khanna P, Clifton AV, Banks D, Tosh GE. Smoking cessation advice for people with serious mental illness. *The Cochrane database of systematic reviews.* 2016;1:Cd009704.

28. McGinty EE, Baller J, Azrin ST, Juliano-Bult D, Daumit GL. Interventions to Address Medical Conditions and Health-Risk Behaviors Among Persons With Serious Mental Illness: A Comprehensive Review. *Schizophrenia bulletin.* 2016;42(1):96-124.

29. Peckham E, Brabyn S, Cook L, Tew G, Gilbody S. Smoking cessation in severe mental ill health: what works? an updated systematic review and meta-analysis. *BMC Psychiatry.* 2017;17(1):252.

30. Roberts E, Eden Evins A, McNeill A, Robson D. Efficacy and tolerability of pharmacotherapy for smoking cessation in adults with serious mental illness: a systematic review and network meta‐analysis. *Addiction.* 2016;111(4):599-612.

31. Banham L, Gilbody S. Smoking cessation in severe mental illness: what works? *Addiction.* 2010;105(7):1176-1189.

32. Mitchell AJ, Vancampfort D, De Hert M, Stubbs B. Do people with mental illness receive adequate smoking cessation advice? A systematic review and meta-analysis. *General hospital psychiatry.* 2015;37(1):14-23.

33. Gilbody S, Peckham E, Bailey D, et al. Smoking cessation for people with severe mental illness (SCIMITAR+): a pragmatic randomised controlled trial. *The lancet Psychiatry.* 2019;6(5):379-390.

34. Boeckmann M, Nohavova I, Dogar O, et al. Protocol for the mixed-methods process and context evaluation of the TB & Tobacco randomised controlled trial in Bangladesh and Pakistan: a hybrid effectiveness-implementation study. *BMJ Open.* 2018;8(3):e019878.

35. Sciences NIoMHaN. How to stop tobacco use-a smart guide. Tobacco Cessation Clinic. In. Bangalore, India2007.

36. Carey RN, Connell LE, Johnston M, et al. Behavior Change Techniques and Their Mechanisms of Action: A Synthesis of Links Described in Published Intervention Literature. *Annals of behavioral medicine : a publication of the Society of Behavioral Medicine.* 2018.

37. Johnston M, Carey RN, Connell Bohlen L, et al. Linking behavior change techniques and mechanisms of action: Triangulation of findings from literature synthesis and expert consensus. . 2018.

38. Michie S, Richardson M, Johnston M, et al. The behavior change technique taxonomy (v1) of 93 hierarchically clustered techniques: building an international consensus for the reporting of behavior change interventions. *Annals of behavioral medicine : a publication of the Society of Behavioral Medicine.* 2013;46(1):81-95.

39. Sarkar BK, West R, Arora M, Ahluwalia JS, Reddy KS, Shahab L. Effectiveness of a brief community outreach tobacco cessation intervention in India: a cluster-randomised controlled trial (the BABEX Trial). *Thorax.* 2016:thoraxjnl-2016-208732.

40. Siddiqi K, Khan A, Ahmad M, et al. Action to stop smoking in suspected tuberculosis (ASSIST) in Pakistan: a cluster randomized, controlled trial. *Ann Intern Med.* 2013;158(9):667-675.

41. Organization WH. Tobacco cessation: a manual for nurses, heealth workers and other health professionals. 2010.

42. Herbeć A, Perski O, Shahab L, West R. Smokers' Views on Personal Carbon Monoxide Monitors, Associated Apps, and Their Use: An Interview and Think-Aloud Study. *Int J Environ Res Public Health.* 2018;15(2):288.

43. Shahab L, West R, McNeill A. A randomized, controlled trial of adding expired carbon monoxide feedback to brief stop smoking advice: evaluation of cognitive and behavioral effects. *Health psychology : official journal of the Division of Health Psychology, American Psychological Association.* 2011;30(1):49-57.

44. Lecrubier Y, Sheehan DV, Weiller E, et al. The Mini International Neuropsychiatric Interview (MINI). A short diagnostic structured interview: reliability and validity according to the CIDI. *European Psychiatry.* 1997;12(5):224-231.

45. West R, Hajek P, Stead L, Stapleton J. Outcome criteria in smoking cessation trials: proposal for a common standard. *Addiction (Abingdon, England).* 2005;100(3):299-303.

46. Snow J, Mann M. Qualtrics survey software: handbook for research professionals. *Qualtrics Labs, Inc.* 2013.

47. Thakur J, Jeet G, Pal A, et al. Profile of risk factors for non-communicable diseases in Punjab, Northern India: Results of a state-wide STEPS survey. *PloS one.* 2016;11(7):e0157705.

48. King BA, Mirza SA, Babb SD. A cross-country comparison of secondhand smoke exposure among adults: findings from the Global Adult Tobacco Survey (GATS). *Tobacco control.* 2013;22(4):e5-e5.

49. Chaiton MO, Cohen JE, McDonald PW, Bondy SJ. The Heaviness of Smoking Index as a predictor of smoking cessation in Canada. *Addictive Behaviors.* 2007;32(5):1031-1042.

50. West R, Hajek P. Evaluation of the mood and physical symptoms scale (MPSS) to assess cigarette withdrawal. *Psychopharmacology.* 2004;177(1-2):195-199.

51. Hurst NP, Kind P, Ruta D, Hunter M, Stubbings A. Measuring health-related quality of life in rheumatoid arthritis: validity, responsiveness and reliability of EuroQol (EQ-5D). *British journal of rheumatology.* 1997;36(5):551-559.

52. EuroQol Group. EQ-5D-5L User Guide: Basic information on how to use the EQ-5D-5L instrument (version 2.0) 2013 04/03/2015. 1990. <http://www.euroqol.org/fileadmin/user_upload/Documenten/PDF/Folders_Flyers/UserGuide_EQ-5D-5L_v2.0_October_2013.pdf>. . Accessed 09/05/2017.

53. Shah S, Ainsworth H, Fairhurst C, et al. Muslim communities learning about second-hand smoke: a pilot cluster randomised controlled trial and cost-effectiveness analysis. *npj Primary Care Respiratory Medicine.* 2015;24(15052).

54. Kroenke K, Spitzer RL. The PHQ-9: a new depression diagnostic and severity measure. *Psychiatric annals.* 2002;32(9):509-515.

55. Löwe B, Decker O, Müller S, et al. Validation and standardization of the Generalized Anxiety Disorder Screener (GAD-7) in the general population. *Medical care.* 2008;46(3):266-274.

56. Gandek B, Ware JE, Aaronson NK, et al. Cross-validation of item selection and scoring for the SF-12 Health Survey in nine countries: results from the IQOLA Project. *Journal of clinical epidemiology.* 1998;51(11):1171-1178.

57. Panamá Y, Guatemala C. ESTANDARIZACION DE METODOS EPIDEMIOLOGICOS CUANTITATIVOS SOBRE EL TERRENO’. 1974.

58. Reynolds NR, Sun J, Nagaraja HN, Gifford AL, Wu AW, Chesney MA. Optimizing measurement of self-reported adherence with the ACTG Adherence Questionnaire: a cross-protocol analysis. *Journal of acquired immune deficiency syndromes (1999).* 2007;46(4):402-409.

59. Browning KK, Wewers ME, Ferketich AK, Diaz P, Koletar SL, Reynolds NR. Adherence to Tobacco Dependence Treatment Among HIV-Infected Smokers. *AIDS Behav.* 2016;20(3):608-621.

60. Hertzog MA. Considerations in determining sample size for pilot studies. *Research in nursing & health.* 2008;31(2):180-191.

61. Teare M, Dimairo M, Shephard N, Hayman A, Whitehead A, Walters S. Sample size requirements to estimate key design parameters from external pilot randomised controlled trials: a simulation study. *Trials.* 2015;2015(15):1-13.

62. Moore GF, Audrey S, Barker M, et al. Process evaluation of complex interventions: Medical Research Council guidance. *bmj.* 2015;350:h1258.

63. Thirsk LM, Clark AM. Using qualitative research for complex interventions: the contributions of hermeneutics. *International Journal of Qualitative Methods* 2017;16(1):1609406917721068.

64. Ritchie J, Spencer L. Qualitative data analysis for applied policy research. *The qualitative researcher’s companion.* 2002;573(2002):305-329.

65. O'Cathain A. *A practical guide to using qualitative research with randomized controlled trials.* Oxford University Press; 2018.
