# Supplementary material for: IMPACT smoking cessation support for people with severe mental illness in South Asia (IMPACT 4S): A protocol for a randomised controlled feasibility trial of a combined behavioural and pharmacological support intervention
Source: PLoS One. 2023 Jun 14;18(6):e0287185. doi: 10.1371/journal.pone.0287185 (PMC10266604; doi:10.1371/journal.pone.0287185)
Supplement: S1 Appendix — (DOCX) [file pone.0287185.s003.docx]

**Appendix 1:** **Safety consideration**

Research procedures: The researcher will provide assurance on participants anonymity, confidentiality and rights on refusing to answer any uncomfortable question, asking for temporary break or stopping the interview altogether and/ withdrawal from study, without any consequence. It is possible for some questions to cause distress. The researcher will seek help from in-house clinical staff (e.g. counsellors) on participant’s behalf, as and when required.

*Behavioural support*: IMPACT 4S is a low risk interventions. However, in the circumstances of anxiety and distress among participants caused by given intervention information or feedback, researchers will seek help from clinical staff on participant’s behalf.

*Pharmacological support*: Bupropion and nicotine replacement therapies are generally safe medicines with few side effects, and are routinely prescribed for smoking cessation including for persons with severe mental illnesses. Nicotine gum will slowly release small amounts of nicotine in the system of participants, but without the harmful chemicals found in smoked tobacco. The IMPACT 4S participants will be informed about likely side effects from bupropion and nicotine gum and would be advised to make immediate contact with the study team following any experience of side effects. A nearest emergency referral services will be facilitated in case of emergency medical needs. Where the participants would be advised to seek help from, with adequate information provided to the health care providers in regard to their trial participation and current medication usage both within and outside the study. Adherence to medications will be documented in the counsellor’s record book during each session. Standard operating procedures (SoPs) on suicidality would be followed to assess and handle levels of risk severity (e.g., low, medium and high risk) of threats to participant’s own life as expressed through thoughts, feelings or other signs. The participants assessed with ‘*Low suicidal risk’* will be recommended for Tele follow up (IVRS- Interactive voice response system) and will be linked with the concerned unit for needed intervention. The participants assessed with ‘*Medium and High suicidal risks’* will be advised to get admitted at emergency facility. With prior consents obtained from the participants, family members will be informed about any heightened risks along with appropriate information provided on monitoring and treatment.
